# Two years of explicit CiTO annotations

**DOI:** 10.1186/s13321-023-00683-2

**Published:** 2023-02-03

**Authors:** Egon Willighagen

**Affiliations:** Department of Bioinformatics-BiGCaT, NUTRIM School of Nutrition and Translational Research in Metabolism, Maastricht University, Maastricht, The Netherlands

## Abstract

Citations are an essential aspect of research communication and have become the basis of many evaluation metrics in the academic world. Some see citation counts as a mark of scientific impact or even quality, but in reality the reasons for citing other work are manifold which makes the interpretation more complicated than a single citation count can reflect. Two years ago, the *Journal of Cheminformatics* proposed the CiTO Pilot for the adoption of a practice of annotating citations with their citation intentions. Basically, when you cite a journal article or dataset (or any other source), you also explain why specifically you cite that source. Particularly, the agreement and disagreement and reuse of methods and data are of interest. This article explores what happened after the launch of the pilot. We summarize how authors in the *Journal of Cheminformatics* used the pilot, shows citation annotations are distributed with Wikidata, visualized with Scholia, discusses adoption outside BMC, and finally present some thoughts on what needs to happen next.

## Main text

Communicating new research findings is still primarily done by written texts in the form of scholarly articles, books, and book chapters. To not having to repeat past research by themselves or others, authors cite relevant research [1]. However, the reasons why authors cite literature vary, which complicates how we use citations [2]. Typing citations is therefore of interest: it allows us to navigate literature more easily: it points us to essential research methods, data, and can warn us of research that cannot be reproduced, or others disagree with [2]. Indeed, it helps us understand the history of science [3].

With the use of citations increasingly being picked up to help researchers with tools like scite.ai [2] en Connected Papers [4], having typed citations will help us explore literature. Therefore, the *Journal of Cheminformatics* started a pilot to explore capturing the intent of citations using annotations [5].

## The Citation Typing Ontology Pilot

The pilot consisted of a couple of components and the editorial explains some of them [5]. The Citation Typing Ontology was selected to express the intention [1], the intention is expressed a compact identifier wrapped in square brackets, also called a safe CURIE, standard proposed by the W3C in 2010 [6, 7]. The *cito* prefix is registered in Bioregistry [8]. The *bibnotes* concept of the Springer Nature publishing platform was used as carrier. Authors are guided by a landing page consisting of a BMC Collection at https://www.biomedcentral.com/collections/cito and author guidelines explaining to authors how they can add the annotations with their favorite editor at https://jcheminform.github.io/jcheminform-author-guidelines/cito.

Because the CiTO ontology has many terms for many different citation intentions, we made a selection of CiTO terms authors could use [9]: **[cito:citesAsDatasource]** to indicate a source that provides data to back up a claim, **[cito:usesDataFrom]** to indicate that the authors reused data, **[cito:usesMethodIn]** when a method or protocol explained in that source is used, and a few more general intentions like **[cito:discusses]**, **[cito:extends]**, **[cito:agreesWith]**, and **[cito:disagreesWith]**. The journal itself would adopt the following CiTO annotations: **[cito:retracts]**, **[cito:repliesTo]**, and **[cito:updates]**. Fortunately, it has not been used yet, but the first would be used if an article was retracted from the journal. The second would be used when a Letter to the Editor replies to an earlier published article, and **[cito:updates]** when a Correction was published.

## Wikidata and Scholia

**Fig. 1:**
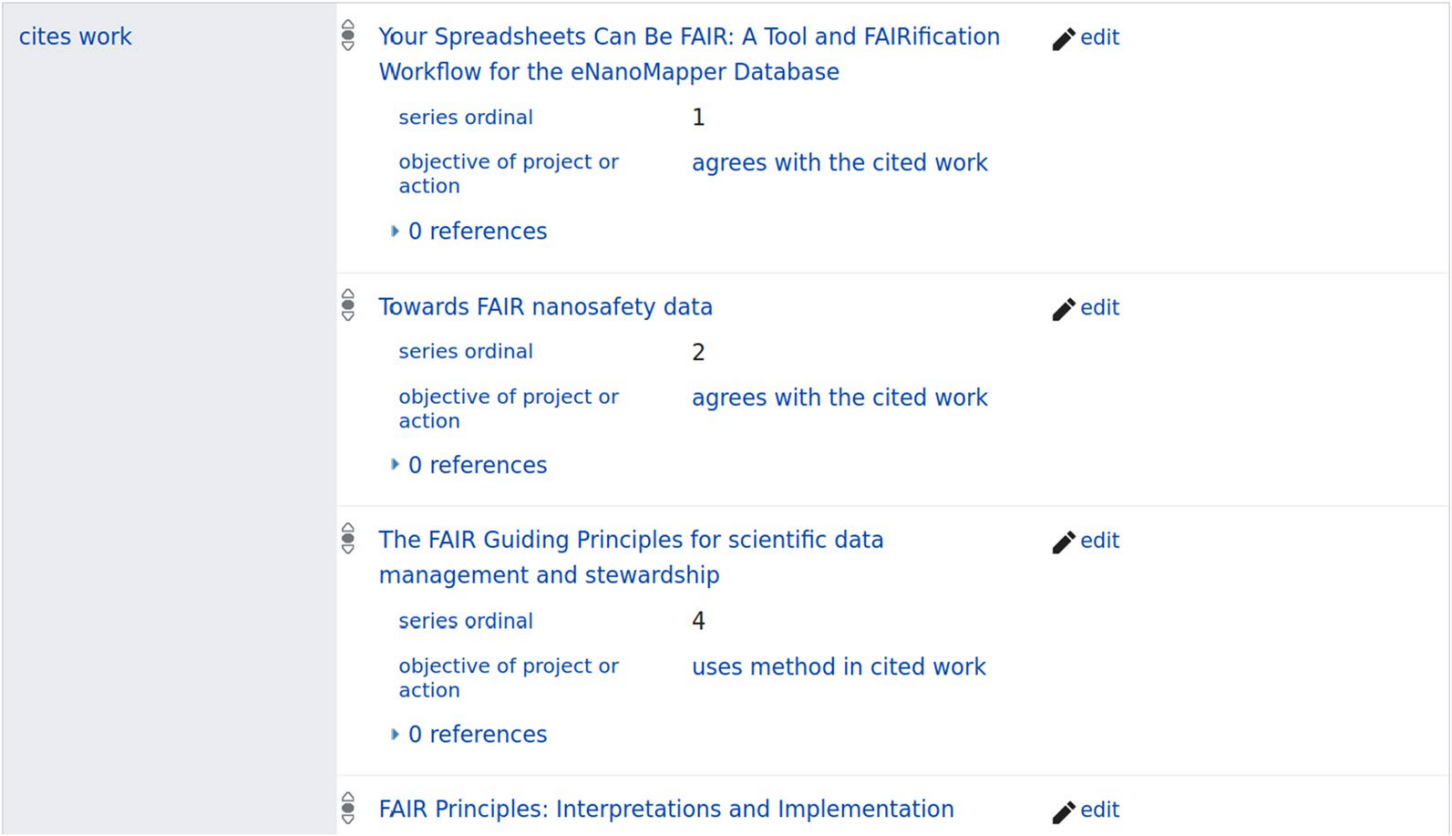
Screenshot of the citation statements for an article where the objective of project or action qualifier is used to annotate the citation with their CiTO intentions

To track the uptake but also to demonstrate the impact, we extended Scholia to visualize citation intention data. Scholia is a graphical interface around the data stored in Wikidata [10] and includes citations from OpenCitations [11] and PubMed. Wikidata allows adding qualifiers to statements which allowed us to define a data model for citations annotated with CiTO intention; the Wikidata property P3712 has been used for this, labeled *objective of project or action* (see Fig. 1; this property was relabeled in November 2022 as *has goal*).

**Fig. 2:**
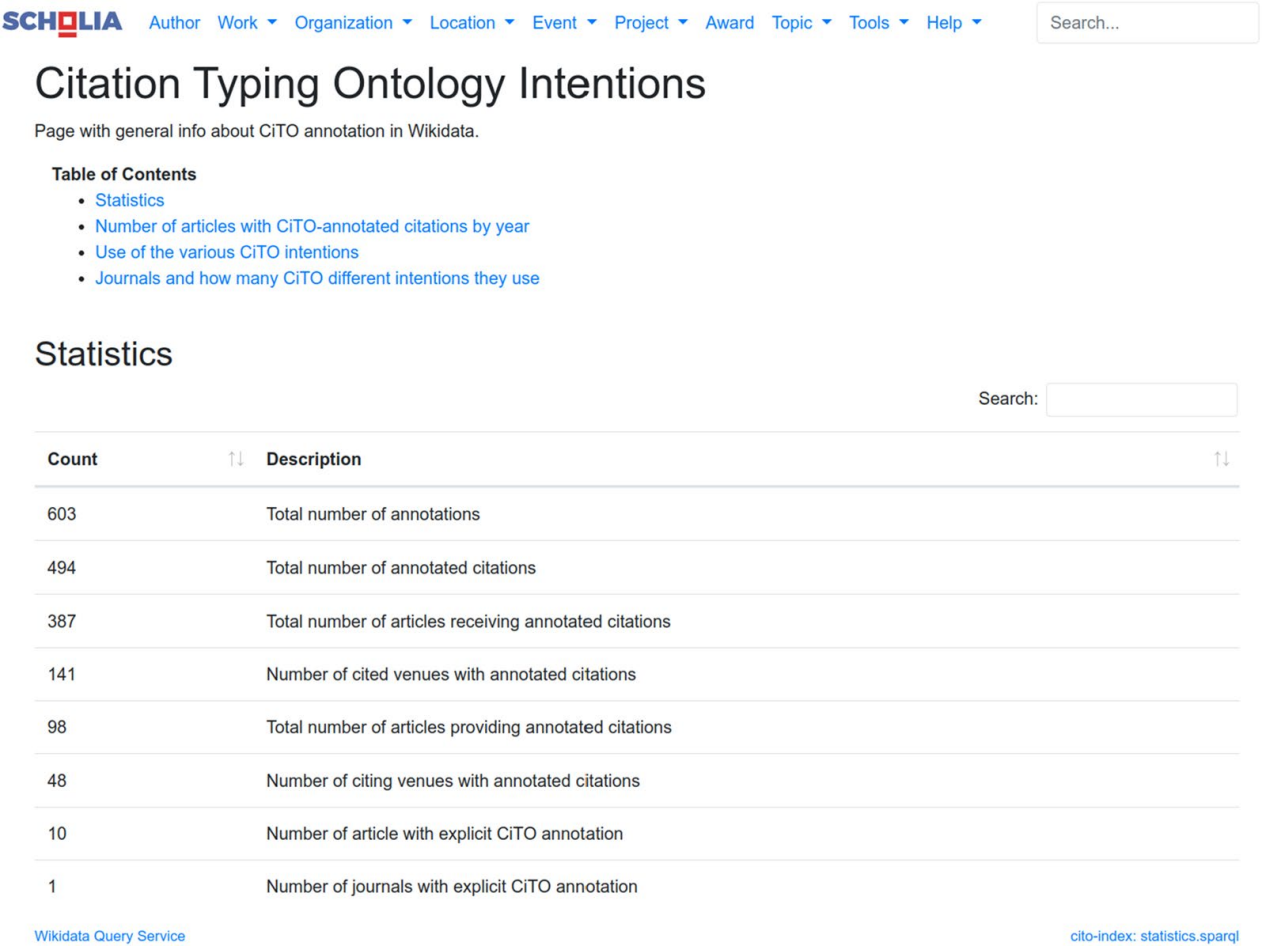
Screenshot of the Scholia Citation Typing Ontology page showing the daily statistics

This data in Wikidata can then be accessed in multiple ways, including REST APIs and a SPARQL interface. The latter is used by Scholia to tell us some overall statistics of the number of annotations, which we reported on about a year ago too [12]. Since last year and recorded on August 25 2022, the number of annotations and the number of annotated citations have almost doubled (from 377 to 603 and from 304 to 494, respectively). The first number is higher because one citation can have more than one citation intention. To continue, the current number of citations are citing 387 articles in 141 different scholarly journals, and they are found in 98 articles in 48 different journals (see Fig. 2) [13].

It must be noted that the *Journal of Cheminformatics* is only one possible source of CiTO annotations. As far as the author knows, it is still the only journal that uses CiTO annotation explicitly in the articles itself. And with 335 annotated citations in 32 articles it also is the major source of CiTO annotations in Wikidata at the time of writing. However, CiTO intention annotations in Wikidata can come from other sources too and be added both manually and automatically using the tools around Wikidata. When all annotation is combined, Scholia shows us that **[cito:citesAsAuthority]** is the most used intention, with 226 annotated citations (out of 603) in 38 articles. **[cito:usesMethodIn]** follows with 102 annotated citations.

## Adoption by *Journal of Cheminformatics* Authors

In the two years of the Pilot, including the seminal editorial [5], the *Journal of Cheminformatics* published fifteen articles with explicit CiTO annotation: three Editorials, four Research articles, two Database, and one of each of Data Note, Software, Letter to the Editor, Letter Response, Educational, and Methodology. Ten were published in the first year (Table 1) and five in the second year (Table 2). Each article annotated one or more citations with CiTO intentions, and several articles annotated every citation, far exceeding the original anticipation.

**Table 1:** Journal of Cheminformatics articles in the first year of the pilot

Type	Article	Intentions	%CiTO
Editorial	Adoption of the Citation Typing Ontology by the *Journal of Cheminformatics* [5]	cito:citesAsAuthority, cito:citesForInformation, cito:usesMethodIn	42%
Methodology	Predicting target profiles with confidence as a service using docking scores [14]	cito:agreesWith, cito:extends, cito:citesAsAuthority, cito:citesAsDataSource, cito:usesMethodIn	47%
Research article	Too sweet: cheminformatics for deglycosylation in natural products [15]	cito:citeAsAuthority, cito:citesForInformation, cito:usesDataFrom, cito:usesMethodIn	
Educational	A ligand-based computational drug repurposing pipeline using KNIME and Programmatic Data Access: case studies for rare diseases and COVID-19 [16]	cito:agreesWith, cito:citesAsAuthority, cito:citesAsDataSource, cito:discusses, cito:usesDataFrom, cito:usesMethodIn	98%
Database	COCONUT online: Collection of Open Natural Products database [17]	cito:citesForInformation, cito:usesDataFrom, cito:usesMethodIn	41%
Editorial	What is the role of cheminformatics in a pandemic? [18]	cito:agreesWith, cito:citesAsAuthority, cito:citesAsDataSource, cito:citesForInformation, cito:citesAsPotentialSolution	91%
Research article	Empowering large chemical knowledge bases for exposomics: PubChemLite meets MetFrag [19]	cito:citesAsAuthority, cito:citesAsDataSource, cito:citesAsMetadataDocument, cito:discusses, cito:extends, cito:usesDataFrom, cito:usesMethodIn	100%
Software	IDSM ChemWebRDF: SPARQLing small-molecule datasets [20]	cito:citesAsAuthority, cito:citesAsRelated, cito:usesDataFrom, cito:usesMethodIn	97%
Letter to the Editor	FAIR chemical structures in the Journal of Cheminformatics [21]	cito:citesAsAuthority, cito:documents	100%
Letter Response	Reply to “FAIR chemical structure in the Journal of Cheminformatics” [22]	cito:agreesWith, cito:citesAsAuthority, cito:citesAsDataSource, cito:obtainsBackgroundFrom, cito:repliesTo	100%

**Table 2:** Journal of Cheminformatics articles in the second year of the pilot

Type	Article	Intentions	%CiTO
Research article	DECIMER 1.0: deep learning for chemical image recognition using transformers [23]	cito:agreesWith, cito:citesAsAuthority, cito:citesAsDataSource, cito:extends, cito:usesMethodIn	66% (*)
Research article	*DrugEx* v2: de novo design of drug molecules by Pareto-based multi-objective reinforcement learning in polypharmacology [24]	cito:extends, cito:citesAsAuthority, cito:usesMethodIn	100%
Database	PSnpBind: a database of mutated binding site protein–ligand complexes constructed using a multithreaded virtual screening workflow [25]	cito:citesAsDataSource, cito:usesDataFrom, cito:usesMethodIn	23%
Editorial	Diversifying cheminformatics [26]	cito:citesAsAuthority, cito:citesAsEvidence, cito:citesForInformation, cito:citesAsRecommendedReading, cito:citesAsSourceDocument, cito:containsAssertionFrom	100%
Data Note	DECIMER—hand-drawn molecule images dataset [27]	cito:agreesWith, cito:cites, cito:citesAsAuthority, cito:extends, cito:usesDataFrom, cito:usesMethodIn	41% (*)

The * indicates that the percentage is based on CiTO intends different from cito:cites

Also exceeding expectations is the diversity of the chosen CiTO intention types. The original guidance focused on **[cito:citesAsDataSource]**, **[cito:usesDataFrom]** (the first is used to cite an article with data, the second when you reused data and cite the article where it comes from), **[cito:usesMethodIn]**, **[cito:citesAsAuthority]**, **[cito:discusses]**, **[cito:extends]**, **[cito:agreesWith]**, and **[cito:disagreesWith]**. Not only have all of these been used, authors also used **[cito:citesForInformation]**, **[cito:citesAsPotentialSolution]**, **[cito:citesAsRelated]**, **[cito:documents]**, and **[cito:obtainsBackgroundFrom]**.

## Technological innovation

To make life easier for authors, and following a Twitter discussion in Spring 2021, a Markdown template was developed with native CiTO support: https://jcheminform.github.io/jcheminform-author-guidelines/cito-guidelines/markdown.html. Here, the author indicates the CiTO type when they cite the article. This is using a method introduced by Krewinkel et al. [28]. The manuscript can then be converted to a Microsoft Word file with Pandoc (https://pandoc.org/) for submission to the journal. For publishers it will be interesting to note that the Pandoc can be directly converted in the *Journal Article Tag Suite* (JATS) format [29, 30].

The *Journal of Cheminformatics* template is available from the journal’s GitHub organization, and authors and editors should feel free to adapt it to the needs of other journals. The BioHackrXiv (https://biohackrxiv.org/) preprint server also support CiTO annotations [31] and this template can be found at https://github.com/biohackrxiv/publication-template.

## Annotated citation networks

We already use citation networks in finding relevant literature, for example based on co-citation patterns. Such analyses become stronger when we know more why articles cited. Similarly, an article that cites an article because it uses a method in second article and that method extends a method in a third articles, then the first article indirectly uses the method in that third article, even if that third article is not directly cited. This is reflecting citation habits: authors always decide whether to cite all articles about a method, only the most recent, or only the oldest (or something else). After all, journals frequently have rules about the maximum length of reference sections. Moreover, some methods are so well established, we are not expected to cite that work at all.

**Fig. 3:**
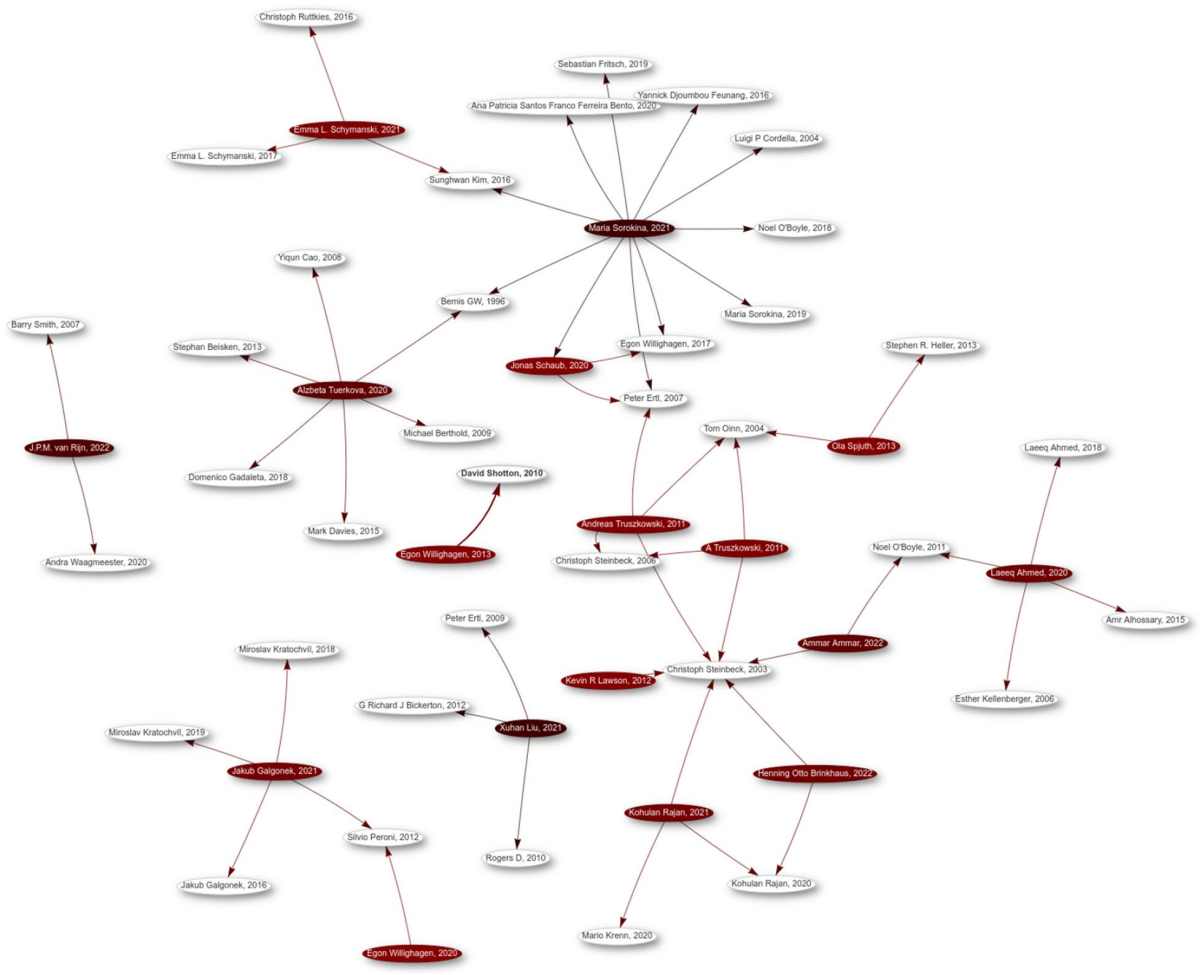
Citation network consisting only of citations annotated with **[cito:usesMethodIn]**. Articles with explicit CiTO annotation are shown in red. The Wikidata SPARQL query used to create this diagram is available from https://gist.github.com/egonw/1c8bc99373a24075838ee19976c74856

The general availability of open citation allows us to recover such more complex reuse scenarios using the citation networks. Moreover, when the citations are annotated, we can zoom in on reuse networks. Figure 3 shows a method reuse network for articles with explicit annotation in red. The network shows a few article that use methods in two cited articles, e.g. *Kohulan Rajan, 2021* and *Henning Otto Brinkhaus, 2022* [23, 27]. Of course, if we do not limit the network to a subset of articles (here, *Journal of Cheminformatics* articles with CiTO annotation), the network becomes more interesting, but also much more complex. Network analysis approaches can then be used instead of network visualization.

**Fig. 4:**
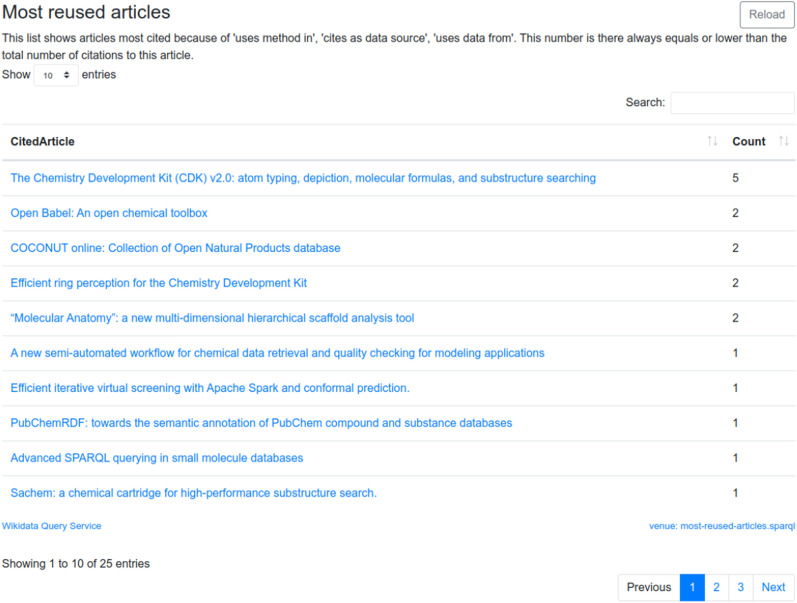
Screenshot of the “Most reused articles” section of the Scholia page for the Journal of Cheminformatics. Based on CiTO annotation available in Wikidata, the ten most reused articles are shown

When we combine the reuse of methods and data, we can for journals summarize which articles are most reused. Analyses like this become a simple query that can be routinely performed for any journal. Figure 4 shows a tabular summary of articles of which methods or data is reused. Like citation counts, this data depends totally on explicit citation data. However, these citation counts are based on actual reuse and not also on the number of citations as authority.

## Discussion

Because many publishing platforms currently do not support display of citation level intention annotation, the simplest model only provides the annotation in the bibliography. The Pilot made this choice to be able to use the bibnotes approach which allows giving additional notes to a reference in the bibliography. The CiTO Pilot uses the *safe CURIE* standard, compatible with the typesetting in the *Journal of Cheminformatics*. This makes is easier to text mine the annotations by downstream tools, from both the HTML and PDF as well as the JATS versions of the article.

However, this links also to the limitation to the current use of CiTO annotation: the citation-level annotation may be supported by some authoring systems (Markdown), even then the depiction may not be supported. If we convert a Markdown file to a Microsoft Word file, the annotation is kept in the bibliography. However, when writing a manuscript in Microsoft Word, LibreOffice, or Google Docs, combined with a reference manager like Zotero, the CiTO annotation cannot be stored as part of the manuscript easily. The problem here is that CiTO annotation in the reference manager are no longer linked to when they are cited. The workaround is to add the CiTO annotation after completion of the Word document, directly to the bibliography.

LaTeX users may find them in a situation between that of Markdown users and reference manager users: only if a manuscript-level Bib(La)TeX file is used the CiTO annotation can be added as notes to the BiBTeX file. This way, the CiTO annotation is specific for this manuscript and each manuscript can use different annotations. This approach is explained in this guidance document: https://jcheminform.github.io/jcheminform-author-guidelines/cito-guidelines/latex.html.

From a use case perspective, it is easy to see how this kind of annotation can be used. For example, we here showed examples of reuse of work, via **[cito:usesMethodIn]** and **[cito:usesDataFrom]**. Second, scite.ai is a clear use case of **[cito:agreesWith]** and **[cito:disagreesWith]** annotation, though it makes a good case how such citation intentions can be extracted with text mining instead. However, other use cases still need development and adoption, which brings us to the question: what is next?

## What is next?

With fifteen articles published in the CiTO Collection, the pilot triggered interest from authors. The *Journal of Cheminformatics* has already published a few more articles with CiTO annotation after August 2022 and a search on the preprint servers ResearchSquare and ChemRxiv [32] show a few more manuscripts. The support by BioHackrXiv [33] is a nice example of adoption beyond BMC. Further citation intention annotations will come from literature studies where citations networks are characterized. For example, Duca et al*.* used CiTO annotations to describe the citation network to retracted COVID-19 articles [34].

Further uptake of this idea of typed citations depends on the combined willingness of journal editors, authors, publishers and indexing services alike. The rise of services like scite.ai shows that the research community is ready for this kind of information. Logical steps forward include support of distributing citation typing annotation via platforms like PlumX, Altmetric.com, CrossRef, or EuropePMC, and support of CiTO annotation in the JATS format.

Because all innovation requires a critical amount of adoption to be accepted, additional sources of CiTO annotation will be welcomed. For example, providing CiTO annotations via a standardized format like a spreadsheet-like format or nanopublications would allow collections of annotations to be shared, such as that by Duca et al. Then, when archived on Zenodo and therefore citable, these annotations can be included in analysis as a trusted or at least citable source.

## Data Availability

CiTO annotation in the *Journal of Cheminformatics* are available from the journal’s articles. CiTO annotation data in Scholia is available from Wikidata. Markdown templates that support CiTO are available from https://github.com/jcheminform/markdown-jcheminf and https://github.com/biohackrxiv/publication-template. An archive of all CiTO annotations in Wikidata is available from https://doi.org/10.5281/zenodo.7513573.
